# Development, Characterization and Stability Evaluation of Topical Gel Loaded With Ethosomes Containing *Achillea millefolium* L. Extract

**DOI:** 10.3389/fphar.2021.603227

**Published:** 2021-04-12

**Authors:** Mehwish Andleeb, Haij Muhammad Shoaib Khan, Muhammad Daniyal

**Affiliations:** ^1^ Pharmacy Department, The Islamia University of Bahawalpur, Bahawalpur, Pakistan; ^2^ Department of Statistics, The Islamia University of Bahawalpur, Bahawalpur, Pakistan

**Keywords:** *Achillea millefolium* L. extract, nanoethosomes, antioxidant, topical drug delivery, statistical approaches

## Abstract

**Background:** Delivering plant extract at high loading with intact antioxidants and efficient skin permeation always remains a challenge. To address this, we prepared a stable gel formulation containing nanoethosomes loaded with *Achillea millefolium* L. (AM) extract for topical drug delivery.

**Method:** The AM extract was tested at first for phytochemical analysis, antioxidant activity, total phenolic and flavonoid content, and FTIR examination. The nanoethosomes containing AM extract were synthesized and characterized by size, surface charge, and morphology, and entrapment efficiency (EE) was determined. The optimized nanoethosomes were then incorporated to develop a topical gel formulation and subjected to skin for permeation, pH, viscosity, and organoleptic evaluation for up to three months.

**Results:** The AM ethanolic extract demonstrated 88% free radical scavenging activity and notable phenolic and flavonoid contents of up to 123 mg GAE/g and 42 mg QE/g, respectively. The optimized nanoethosomes encapsulated with AM extract (240 nm) were spherical in shape, with −31.1 mV of surface charge, and showed considerable entrapment efficiency (90%). Furthermore, the selected topical gel remained stable during the study period. The Exvivo permeation study of ethosomal gel showed the highest release percentage of 79.8%.

**Conclusion:** The study concludes that topical gel loaded with nanoethosomes containing AM extract is an encouraging approach for topical drug delivery.

## Introduction

It is well known that cardiovascular diseases, cancer, and neurodegenerative diseases, especially the aging process are due to the production of free radicals. Because antioxidants are capable of neutralizing these free radicals, there is a need to discover new sources of them (Ratnam et al., 2006). Various medicinal plants have been investigated for their antioxidant activity. In this case, the natural antioxidants both in extract form or chemical constituents could be employed to avoid free radical associated oxidative stress (Saeed et al., 2012). Flavonoids are recognized for their free radical scavenging and antioxidant properties. The position of the substituents also affects the physiological roles of different flavonoids. Flavonols from the ortho or para hydroxyl group within the two- phenyl rings have sturdy antioxidant residences, and unfastened hydroxyl on the 5,7-positions was proven to have a seasoned-oxidant impact (Eghdami and Sadeghi, 2010).

*Achillea millefolium* L. belongs to the Asteraceae family and its miles are represented by means of approximately 85 species ordinarily located in Europe and Asia and a handful in the northern United States. Its extract is widely used for the treatment of fever, asthma, bronchitis, cough, pores, skin irritation, jaundice, diabetes, hepatobiliary illnesses, wound healing, menstrual pain, flatulence, dyspepsia, hemorrhoids, dysmenorrhea, gastritis and is also used for its antitumor, antimicrobial, and antioxidant properties (Mohammadhosseini et al., 2017). Previously, numerous studies have been carried out to explore its phytochemical composition, which identified flavonoids and caffeic acid derivatives. A majority of these studies enhanced understanding of the chemical composition of these species, making dermal delivery a promising choice (Vitalini et al., 2011).

The outermost layer of skin (stratum corneum) consists of corneocytes enclosed by the lipid bilayers. This is the main barrier to deliver the drug to the skin. Many techniques are being used to overcome the problem, including ethosomes. The composition of ethosomes is based on phospholipids and ethanol (20–40%) already developed by Touitou et al., 2000. Ethanol is used as a skin permeation enhancer, allowing it to transport through the skin easily. Studies have already shown the effectiveness of ethosomes across the skin in terms of depth and quantity (Baek et al., 2015; Limpongsa et al., 2015).

In this study, *Achillea millefolium* L. plant extract was successfully encapsulated in nanoethosomal vesicles, followed by its incorporation in gel for topical delivery. The optimized topical gel is meanwhile loaded with nanoethosomes, enclosing AM extract was formulated by placing them at different temperatures for 3 months and characterized by organlopetic evaluation, pH, and viscosity.

## Materials and Methods

The Methanol, ethanol, n-Hexane, and Propylene glycol were purchased from Merck, Germany. 1,1-Diphenyl-2-picrylhydrazyl and TRITON X-100 were obtained from Sigma, United States, and Phospholipid was gifted by LIPOID (Switzerland). The size, surface charge, and Entrapment efficiency of ethosome vesicles containing AM extract was determined by SEM and ultracentrifugation, respectively. The Fourier Transform Infrared Spectroscopy (FTIR) was performed for the analysis of functional groups of AM extract loaded ethosomes.

### Extraction of *Achillea millefolium* L.

The *Achillea millefolium* L. was obtained and authenticated from the Center of Biodiversity and Conservation Shah Abdul Latif University, Khairpur, Sindh- Pakistan (reference no. CBC/SALU/Khp482).

Then air-dried antenna parts of *Achillea millefolium* L*.* were extracted with 70% ethanol. The maceration was carried at room temperature for 72 h. The extract was then filtered on filter paper and the process was repeated three times. Subsequently, the hydro-alcoholic extract was concentrated up to 1/3 of its initial volume using a rotary vacuum evaporator at 40°C, and the pressure was reduced. The final dark-colored extract was stored at 8°C.

### Analysis of Extract

#### DPPH Free Radical Scavenging Activity

The free radical scavenging activity was determined by 2-diphenyl-1-picryl-hydrazyl (DPPH), as previously described with slight modification (Ratshilivha et al., 2014).

Ascorbic acid was used as standard and percentage inhibition was determined by the following formula.

$$\mathrm{Inhibition}\left(\%\right)=\frac{\mathrm{Absorbance}\;\mathrm{of}\;\mathrm{test}\;\mathrm{solution}}{\mathrm{Abs}.\;\mathrm{of}\;\mathrm{control}}\times 100,$$

where,

Absorbance of control = total radical activity without inhibitor.

Absorbance of test = activity in the presence of test compound.

### Phytochemical Screening

In order to identify the phytochemicals in the extract (flavonoids, carbohydrates, saponins, glycosides, tannins, alkaloids, and proteins), a phytochemical analysis was performed. Briefly, Dragondroff’s reagent, Mayer’s reagent, Hager’s reagent, and Wagner’s reagent were used to detect the alkaloids. Lead acetate test, Ferric chloride test, Froth test, potassium hydroxide test, Ninhydrin test, Biuret test, Anthraquinone glycoside test, Molisch’s and Benedict’s test, Libermann-Burchard reaction, Phenol test, Salkowski’s and LibermannBurchard’s test were used to detect the flavonoids, tannins, saponins, fixed oil and fats, proteins and amino acid, glycosides, reducing sugars, steroids, phenols, diterpenes, and photosterols, respectively (Rahman, 2013; Tiwari et al., 2011).

### Total Phenolic and Flavonoids Contents

The Folin Ciocalteus method was used to determine the total phenolic contents and results were expressed as milligram gallic acid equivalent per gram of extract (mg of GAE/g extract). Similarly, the aluminum chloride colorimetric method was utilized for the determination of total flavonoid content (TFC). The results of TFC were described as milligrams of quercetin equivalent per gram of extract (mg QE/g extract).

### FTIR Analysis

Fourier Transform Infrared Spectroscopy (FT-IR) was performed to detect the active functional groups in the extract by Infrared spectrophotometer (Tensor 27, Bruker Optics GmbH, Ettlingen, Germany). Before obtaining the IR spectrum, the dried samples milled with potassium bromide and 300 kg/cm^2^ pressure was applied to the mixture to form a pallet. The KBr disks were used to develop the spectrum over a range of 4,000–400 cm^−1^.

### Preparation of AM Extract Loaded Nanoethosomes

The nanoethosomes were prepared by a simple cold method. Briefly, lipid (1–3%) was dissolved in a mixture of ethanol (20–40%) and propylene glycol (20%) in a completely closed flask using a magnetic stirrer at 30°C. Next, the AM extract (2%) was introduced slowly as a fine stream with the help of a syringe and the volume was adjusted with distilled water. The whole system was stirred for 15 min at 900 rpm, followed by sonication for 30 min. The nanoethosomes loaded with AM extract were then stored at room temperature. A total of nine batches (AM1-AM9) of ethosomes were prepared by changing the concentration of ethanol and lipid, as described in Table 1.

**TABLE 1:** Composition of ethosomes formulations.

Formulation code	SPC % (w/w)	Ethanol % (w/w)	Water % (w/w)	Extract % (w/w)	Propylene glycol % (w/w)
AM1	1	20	57	2	20
AM2	2	30	46	2	20
AM3	3	40	35	2	20
AM4	1	20	57	2	20
AM5	2	30	46	2	20
AM6	3	40	35	2	20
AM7	1	20	57	2	20
AM8	2	30	46	2	20
AM9	3	40	35	2	20

SPC: Soyaphosphatidylcholine.

## Characterization of Nanoethosomes

### SEM Examination

The surface morphology of the nanoethosomes was observed through Scanning Electron Microscopy (SEM). Prior to analysis, the ethosomal samples were mounted onto double-sided tape that had previously been secured on copper stubs and coated with platinum, then analyzed at different magnifications.

### Determination of Particle Size, PDI, and Zeta Potential

The vesicle size and PDI of nanoethosomes were measured by using Zetasizer (Malvern Instruments, United Kingdom) equipped with software (version 6.34).

### FTIR Analysis

The FTIR analysis of nanoethosomes was carried out as described above.

### Entrapment Efficiency

The entrapment efficiency (EE) of nanoethosomes was calculated by the ultracentrifuge method. Briefly, nanoethosomes were kept overnight at 4°C and centrifuged in an ultracentrifuge at 12,000 rpm for 30 min. Then the supernatant was collected, diluted with water and drug concentration was determined at 290 nmin both vortex and non-vortex samples. Finally, the EE was calculated by the following equation.

$$\mathrm{EE}\left(\%\right)=\frac{\mathrm{Amount}\;\mathrm{of}\;\mathrm{AM}\;\mathrm{in}\;\mathrm{sediment}}{\mathrm{Total}\;\mathrm{amount}\;\mathrm{of}\;\mathrm{AM}\;\mathrm{added}}\times 100.$$

### Formulation of Nanoethosomal Topical Gel

Among nine batches, the optimized formulation AM nine was incorporated into the gel. The Carbopol 940 (carboxyvinyl polycarbomer) was soaked in distilled water for 1 h followed by the addition of 10 ml of nanoethosomes and continuously stirred at 700 rpm at 30°C in a closed vessel until a homogeneous gel was obtained. Finally, Triethanolamine was added drop by drop to adjust the pH of the gel.

### Skin Permeation Studies

*In-vitro* permeation studies were performed for different combinations including ethanolic extract, nanoethosomes, conventional topical gel, and gel having nanoethosomes containing the extract. Studies were carried out in Franz Diffusion cell. Prior to the experiment, fresh rat skin was clamped between the donor and receptor compartments of the vertical cell. The receptor compartment was filled with PBS (pH 7.4) at 32°C under constant stirring at 250 rpm for 24 h. Next, the formulation was applied in the donor compartment. Aliquots of 1 ml were collected at a predetermined time and the medium was replenished with 1 ml fresh medium. Then the samples were diluted and analyzed by spectrophotometer. Finally, cumulative drug permeated per unit area was plotted against time whereas; the slope of the linear portion indicated the transdermal flux.

### Characterization of Topical Nanoethosomal Gel

The prepared nanoethosomal topical gel was further characterized for organlopetic, pH, and viscosity evaluation at 4, 25, and 40°C for three months (day 1, 7, 15, 30, 60, and 90), using a Digital pH meter and Brookfield DVIII Ultra Rheometer, respectively.

### Ethical Approval

The rat skin permeation experiment was approved by the animal ethical board committee at The Islamia University of Bahawalpur, Pakistan, and followed local, national, ethical, and regulatory principles (number designated, PAEC/20/32).

### Statistical Analysis

De-sign-Expert 8.0.6.1 software (Stat-Ease Inc., Minneapolis, Min, United States) was used to optimize the statistics. Results were described as mean ± SD. The statistical analysis was performed using a student t-test and the significance of the difference is indicated as *p < 0.05.

## Results

### DPPH Free Radical Scavenging Activity

In the present study, the free radical scavenging activity of extract of *Achillea millefolium* L. antenna parts was determined by using the DPPH method. The plant showed 88.6 ± 0.17% antioxidant activities as compared to the standard i.e. Ascorbic Acid.

### Phytochemical Analysis

Various phytochemical tests were performed on the extract of antenna parts of *Achillea millefolium* L. to detect the presence of secondary metabolites as shown in Table 2. The formation of green-colored and white-colored precipitates with the respective reagents confirmed the presence of tannins in the samples. All other metabolites of AM extract were identified in the study conducted by using the method adopted by Souza et al., in 2006 (Souza et al., 2006).

**TABLE 2:** Phytochemical analysis of extracts.

S. No.	Secondary Metabolites	AM
1.	Tannins	+
2.	Flavonoids	+
3.	Phenols	+
4.	Saponins	+
5.	Alkaloids	+
6.	Terpenoids	+
7.	Carbohydrates	+
8.	Reducing sugar	−
9.	Steroids	+
10.	Glycosides	+

+ Indicates presence of metabolites in sample; − indicates absence of metabolite in sample.

### Total Phenolic and Flavonoids Contents

The value of total phenolic contents of *Achillea millefiolium* L. were 123.87 ± 0.21 mg GAL/g and total flavonoid contents of selected plant extract were 42.10 ± 0.29 mg QE/g.

### Fourier Transform Infrared Spectroscopy Analysis

**FIGURE 1:**
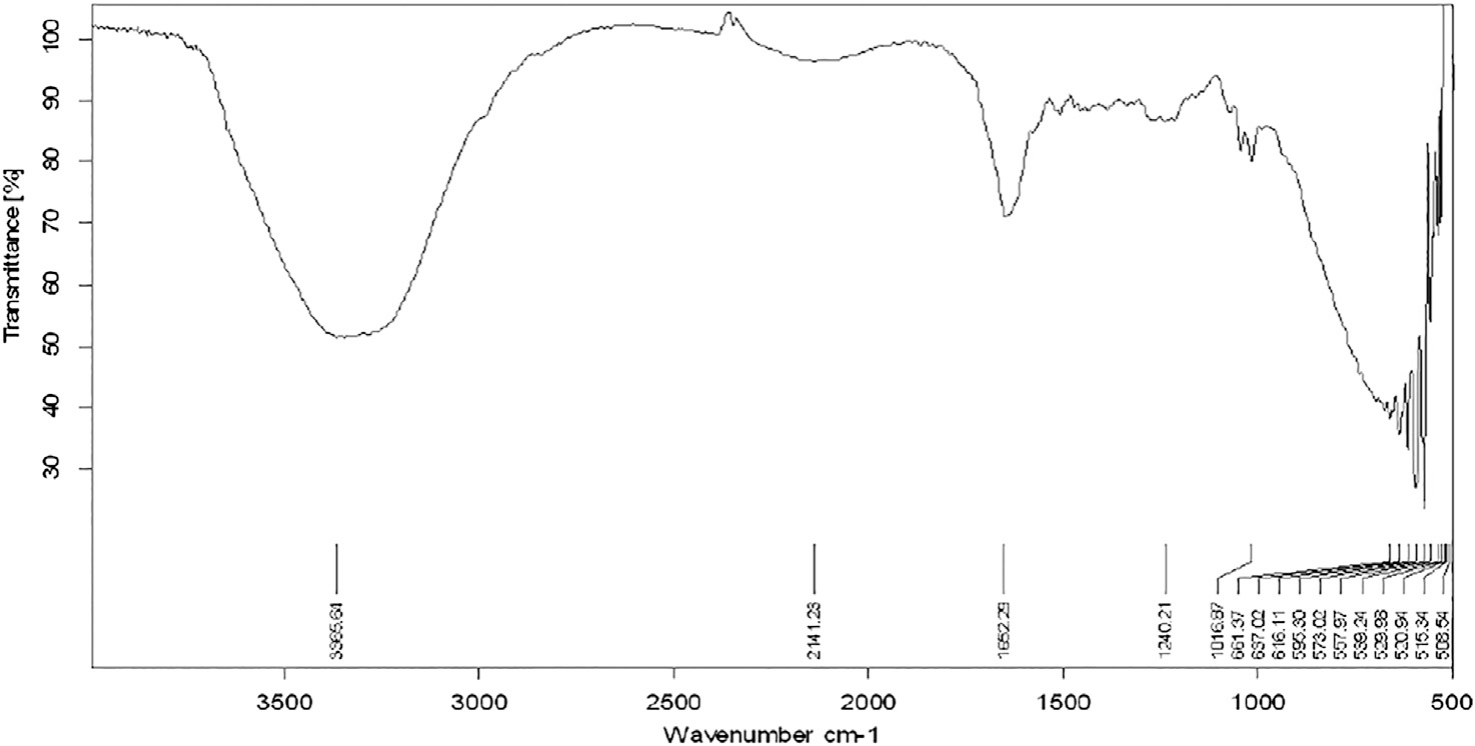
FT-IR spectra of *Achilleamillefolium* extract.

**FIGURE 2:**
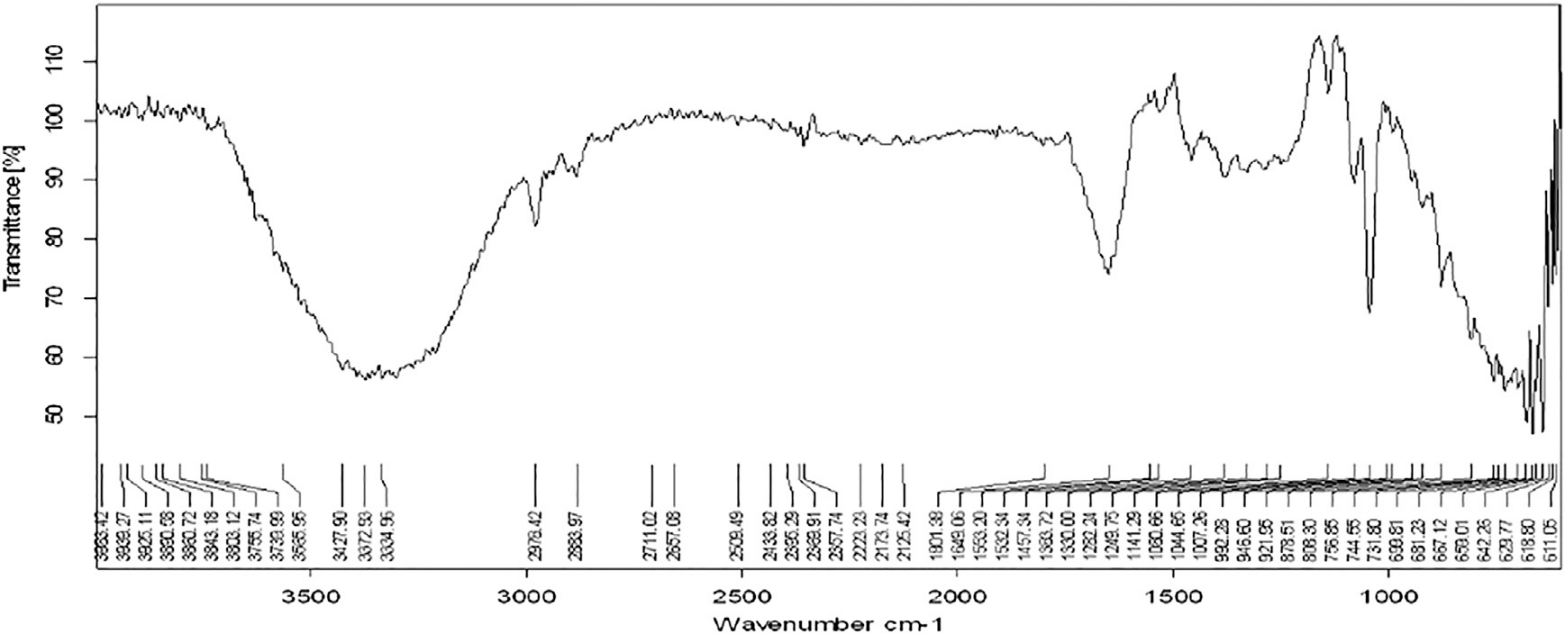
FTIR spectra of herbal ethosomes.

The functional groups present in the plant extract and herbal Ethosomal system were detected by using an FT-IR spectrophotometer. The spectral interpretation of each sample is given in Table 3 and graphically represented in Figures 1, 2. The major peaks that appeared in the herbal ethosomal system were almost the same as those in the pure ethanolic extracts indicating no significant interaction in the herbal extracts and other excipients in the dispersed system.

**TABLE 3:** Spectral interpretation of FTIR spectra of plant extract.

Sr#	Plant extract	Peak values	Functional group	Possible secondary metabolites
1	*Achillea millefolium*	3365.64	O-H (alcohol)	Alkaloids, flavonoids, tannins, saponins, polyphenols, carbohydrates, steroids, terpenoids, carboxylic acid containing phytochemicals etc.
		2141.23	C≡N (nitriles)	
		1662.29	C-0(carbonyl)	

Figure 1 shows the FT-IR spectra of extracting antenna parts of AM. The peak that appeared at 3365.64 cm^−1^ indicates the présence of alcohols and phenols in the extract. The peak that appeared at 2141.23 cm^−1^ indicates the presence of nitriles while the peak at 1,662 points indicates the presence of the carbonyl group in the sample.

### Ethosomes Vesicles Visualization

**FIGURE 3:**
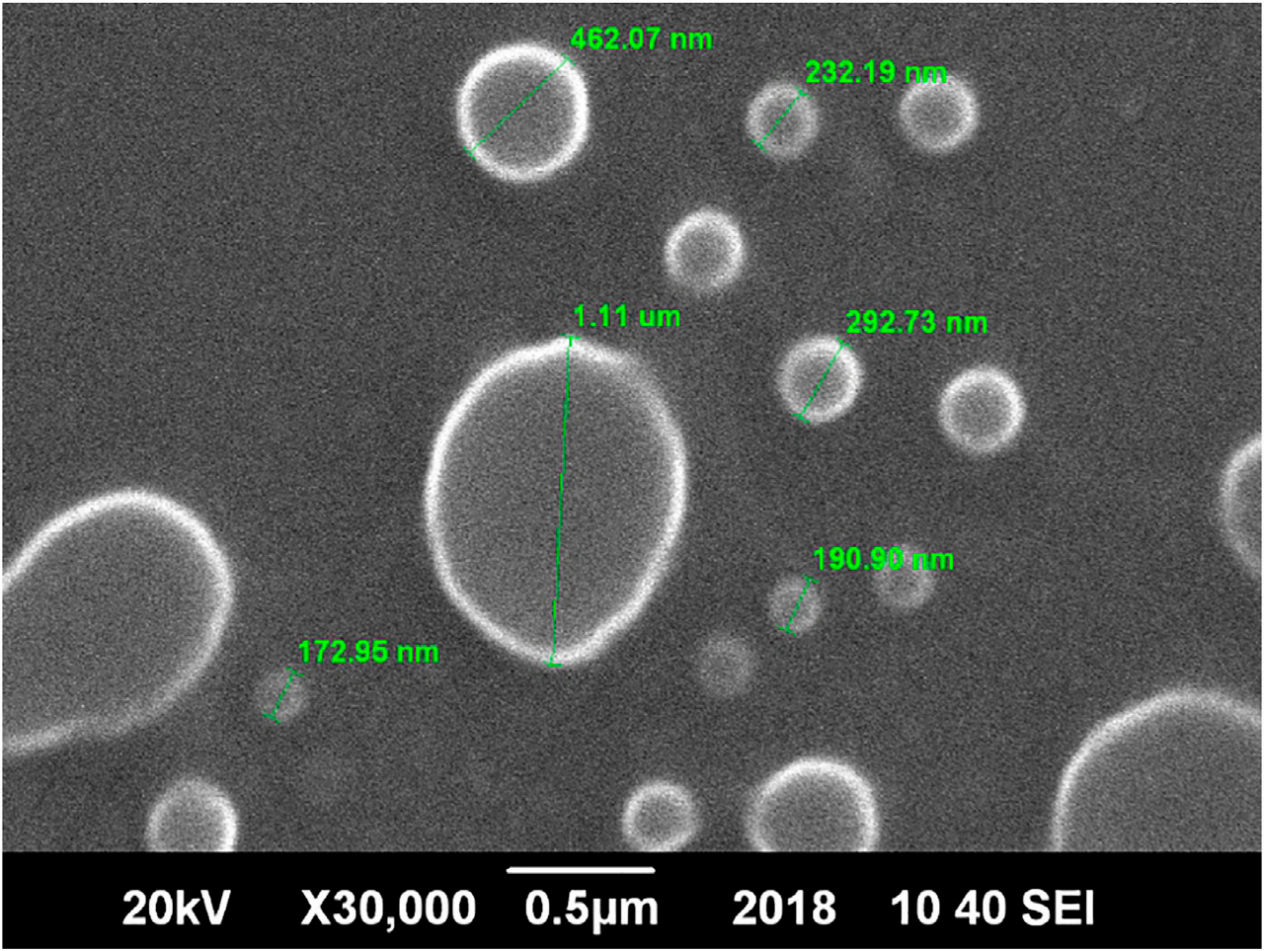
SEM micrograph of optimized herbal ethosomes.

SEM analysis of the optimized ethosomes showed smooth, unilameller, nano sized spherical vesicles Figure 3.

### Vesicle Size

**FIGURE 4:**
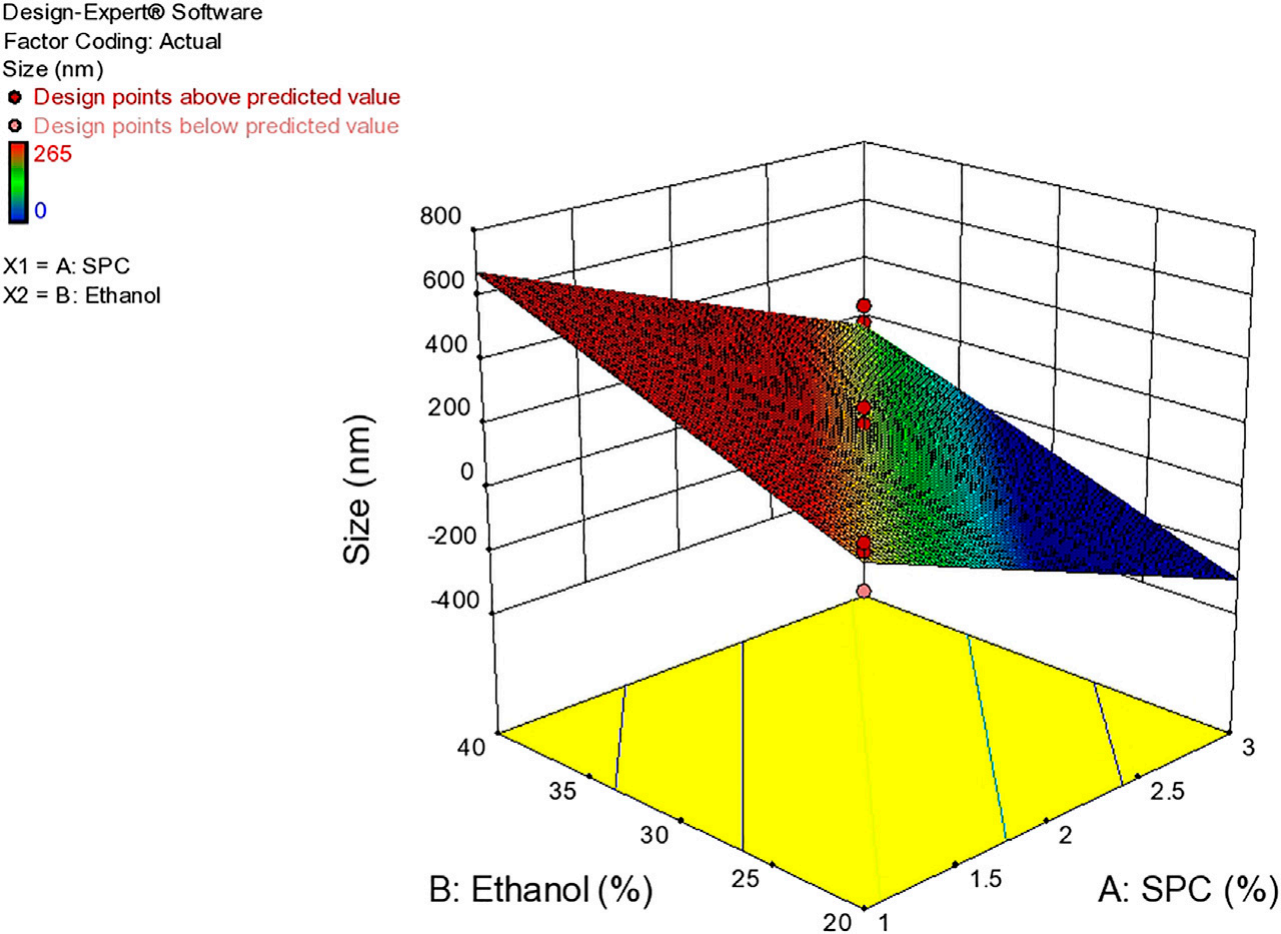
Response surface plots showing effect of lipid and ethanol on size.

**FIGURE 5:**
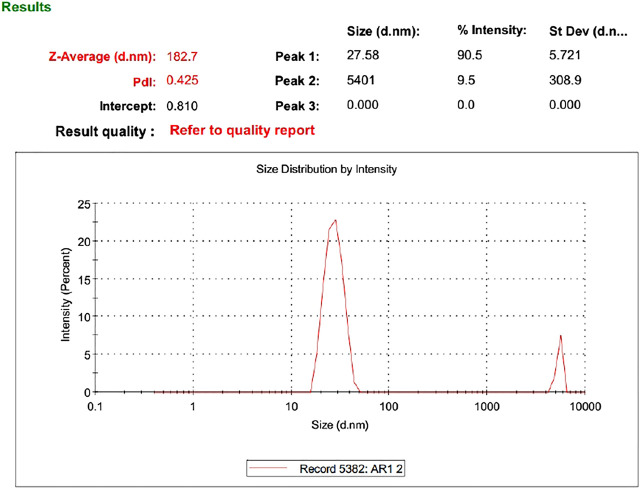
Size of optimized formulation.

As evident from the 3-D and contour plotthat, as the concentration of ethanol increases, the size of the vesicles increases, while the SPC has an inverse relation with the size of particles in the dispersed system, as shown in the response surface, graph Figure 4, while the size of the optimized formulation is given in Table 4 and graphically shown in Figure 5.

**TABLE 4:** Effects of variable on responses.

Formulation code	Entrapment efficiency %±SD	Size (nm)	Polydispersity index ±SD	Zeta potential (mV)
AM1	58.17 ± 1.53	130	0.81 ± 0.050	−37.9
AM2	66.8 ± 1.14	115	0.93 ± 0.048	−31.5
AM3	73.95 ± 0.70	98	1.2 ± 0.042	−38.6
AM4	72.1 ± 1.97	240	0.6 ± 0.016	−34.8
AM5	81.45 ± 2	206	0.58 ± 0.052	−35.3
AM6	84.16 ± 1.82	182	0.42 ± 0.003	−31.5
AM7	86.12 ± 1.83	265	0.46 ± 0.013	−23.2
AM8	88.1 ± 1.30	258	0.48 ± 0.016	−21.8
AM9	90 ± 0.74	240	0.24 ± 0.017	−31.1

The results are mean ± SD (*n* = 3), SD: Standard deviation.

### Zeta Potential

**FIGURE 6:**
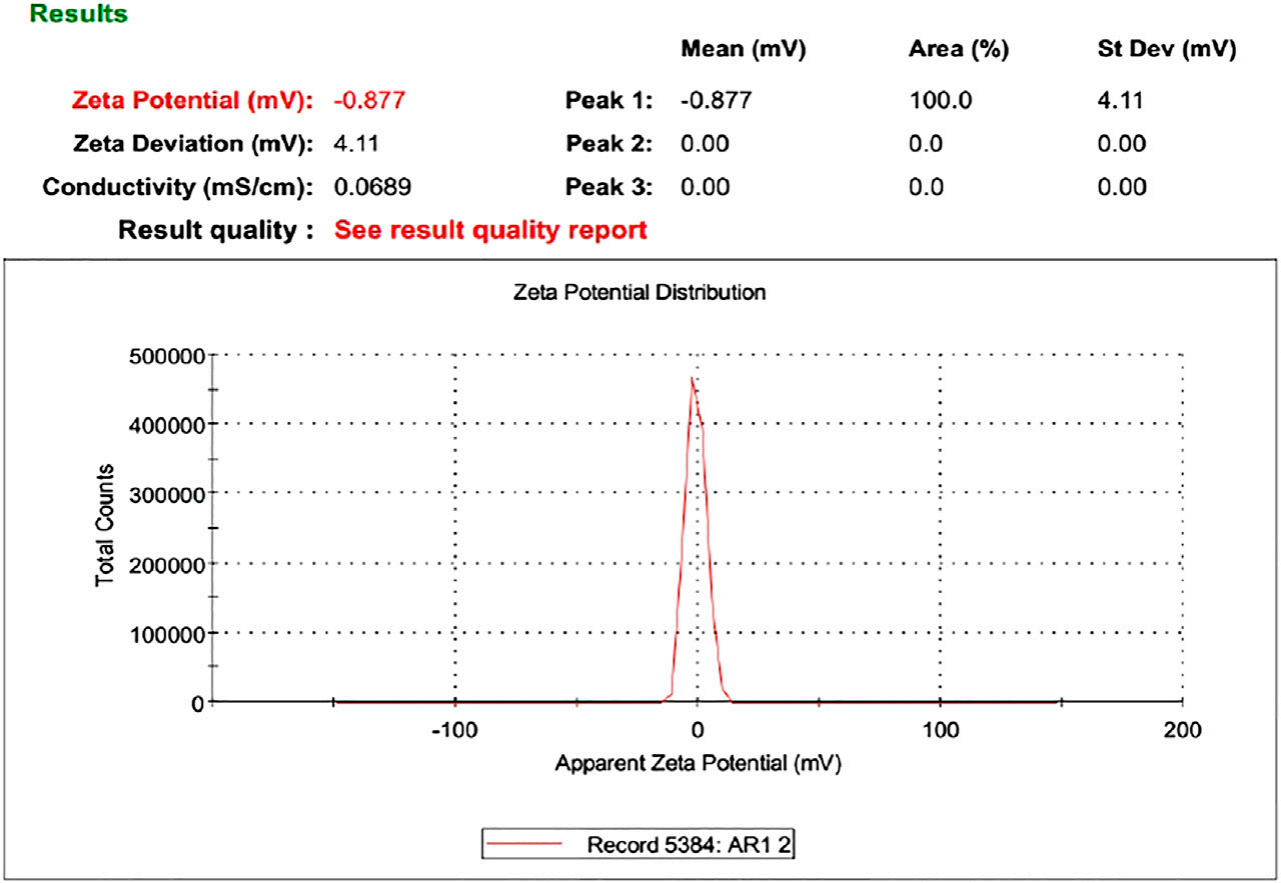
Zeta potential of AM.

**FIGURE 7:**
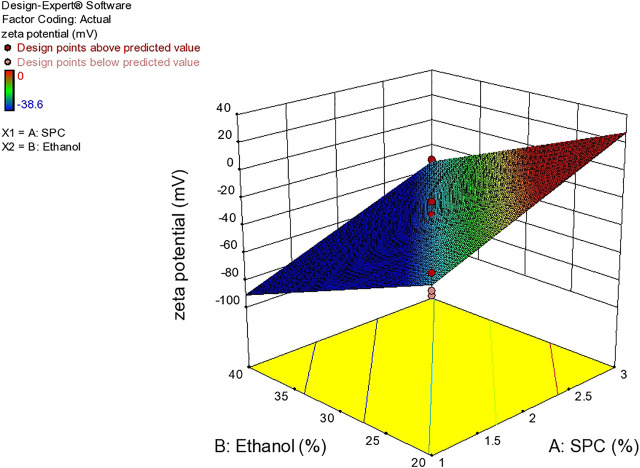
Response surface plots showing effect of lipid and ethanol on Zet potential.

Zeta potential is an important parameter that affects the stability of the drug. The value of zeta potential ranges from −30 mV to 30 mV as shown in Table 4 and Figure 6. The response surface graph shown in Figure 7 shows that, with an increase in ethanol concentration, zeta potential values get negative while SPC has an inverse relation. The enhanced permeation is achieved by negative zeta potential. As shown in Table 4, ZP values of all formulations are negative indicating a stable ethosomes vesicle (Ascenso et al., 2015).

**FIGURE 8:**
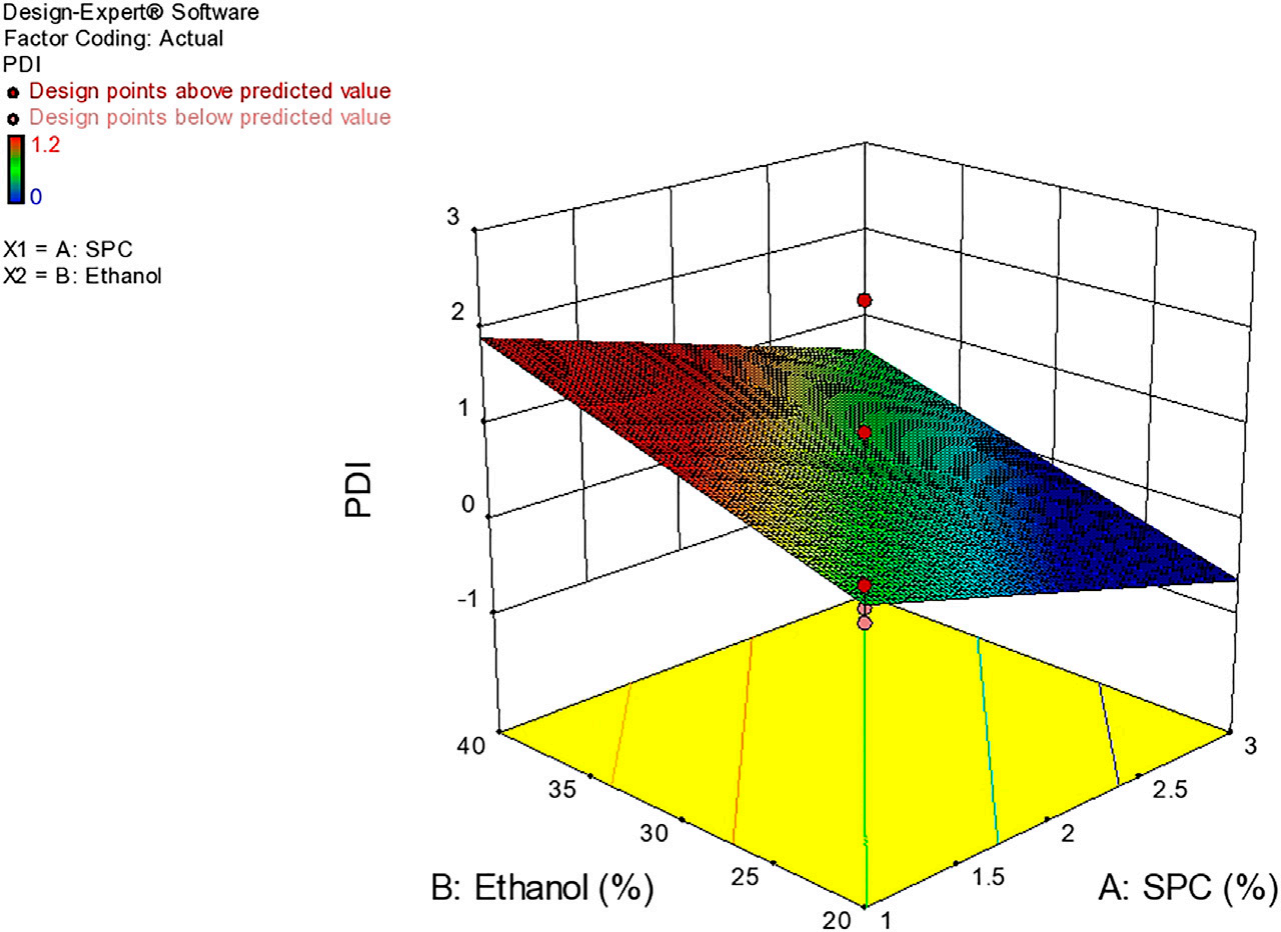
Response surface plot showing effect of ethanol and lipid on PDI.

### Polydispersity Index

**FIGURE 9:**
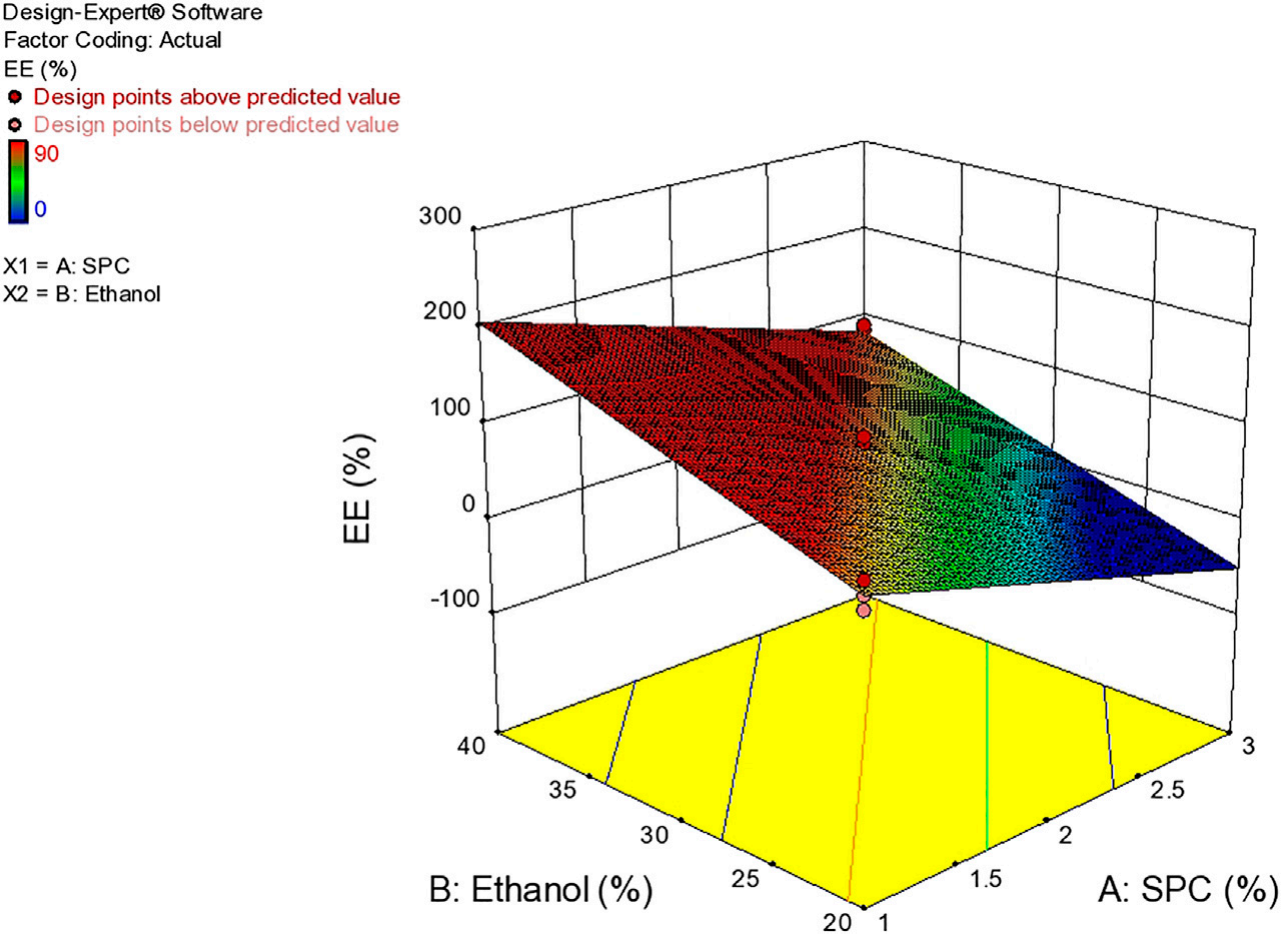
Response surface plots showing effect of lipid and ethanolon EE.

Based on vesicle size distribution, PDI was considered for the homogeneity of the ethosome vesicle preparations. The values of PDI of the selected formulation were 0.24 ± 0.017 (Table 4). The design expert shows the relationship between ethanol and SPC through the response surface graph Figure 9.

### Entrapment Efficiency

The delivery potential is directly determined in terms of the entrapment efficiency of ethosomal dispersion. The Entrapment Efficiency of all the formulations was determined as shown in Table 4. The formulation prepared with 3% SPC and 40% ethanol showed maximum entrapment efficiency (90%). This behavior may be due to the presence of a higher concentration of ethanol which increases AM solubility in ethosomes (Garg et al., 2016) Figure 9.

### *Ex Vivo* Permeation Studies

The cumulative amount of drug is released from *in vitro* diffusion studies of ethanolic extract, ethosomal formulation, conventional gel, and ethosomal gel were found to be 30, 78.6, 28.7 and 79.8%, respectively.

### Topical Gel Characterization

The optimized formulation of AM nine topical gel loaded with ethosomes containing AM extract was evaluated for organoleptic evaluation (color, odor, liquefaction, phase separation), pH, and viscosity.

### Organoleptic Evaluation

In the due time of stability studies, the organoleptic characteristics and homogeneity remained unaffected at different temperatures. All the formulations remained light yellow but changed to a dark color at a higher temperature (Table 5).

**TABLE 5:** Organoleptic evaluation of topical gel at different temperature for 90 days.

Parameter	Temp. (°C)	0 h.	24 h.	7 days	15 days	30 days	60 days	90 days
Color	4	LY	LY	LY	LY	LY	LY	LY
	25	LY	LY	LY	LY	LY	LY	LY
	40	LY	LY	Y	Y	Y	DY	DY
	—							
Odor	4	—	—	—	—	—	—	—
	25	—	—	—	—	—	—	—
	40	—	—	—	—	—	—	—
Liquefaction	4	—	—	—	—	—	—	—
	25	—	—	—	—	—	—	—
	40	—	—	—	—	—	—	—
Phase separation	4	—	—	—	—	—	—	—
	25	—	—	—	—	—	—	—
	40	—	—	—	—	—	—	—

LY = Light yellow; Y=Yellow; DY = Dark yellow; — = No change.

### pH and Viscosity

**FIGURE 10:**
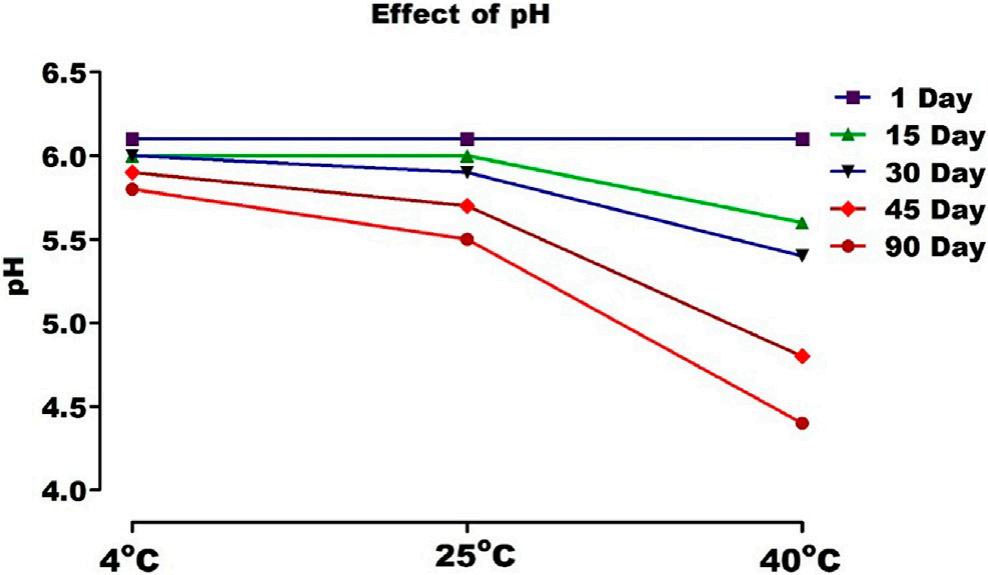
pH of topical gel containing AM extract at different temperature.

The pH values of the gel stored at 4, 25, and 40°C were from 6.1 to 5.5, as shown in Figure 10, which is near to skin pH.

## Discussion

The results suggest that AM extract can be used in various pharmaceutical and cosmeceutical formulations due to its strong antioxidant properties. The natural compounds including the phenolic compound in these extracts are well known for their use in cosmetics (Keser et al., 2013). Phenols are responsible for the determination of redox activities and act as antioxidants. Previous studies have also shown a higher phenolic content in the sample, which indicated the antioxidant potential of the plant. Flavonoids have proved a highly effective scavenger for most oxidizing molecules and various free radicals. They are regarded as the chief contributors in the antioxidant activities of plant material (Eghdami and Sadeghi, 2010). It can be said that the antimicrobial, anti-inflammatory, antifungal, and antioxidant activities of plants are due to secondary metabolites such as tannins and flavonoids in which the hydroxyl group is the key functional component (Kähkönen et al., 1999). The functional groups in samples are also responsible for determining physicochemical properties such as acid-base nature, solubility, and partition coefficient, etc. (Poojary and Passamonti, 2015).

In the present study, FT-IR analysis of the selected plant extract revealed the presence of the most important bioactive biomolecules. Similar studies have also confirmed the presence of various bioactive molecules in the plant extract of *Bauhinia racemosa*, *Eclipta alba*, and *Eclipta prostrate*. In all these plants the results of FT-IR analysis confirmed the presence of saponins, resins, tannins, flavonoids, and phenolic compounds (Liu and Ng, 2000 *;* Muruganantham et al., 2009).

It is evident that if the particle size of the vesicle is less than 300 nm, they can deliver their cargo into a deeper layer of skin. It is evident from the study that the size of the vesicle depends mainly on the different concentrations of lipid and ethanol present in the formulation (Sahu and Bothara, 2015).

As reported in the literature, the particle size should be smaller than 300 nm to ensure efficient skin penetration. A higher concentration of ethanol results in a reduction in thickness of membrane vesicles, and hence efficient penetration into the deep skin layer (Ascenso et al., 2015). PDI values greater than 0.7 show the presence of larger particles in the ethosomal dispersions and indicate the heterogeneous system. In the present system, most of the values fall in the criteria indicating a homogenous system with narrow particle size distribution (Danaei et al., 2018).

**FIGURE 11:**
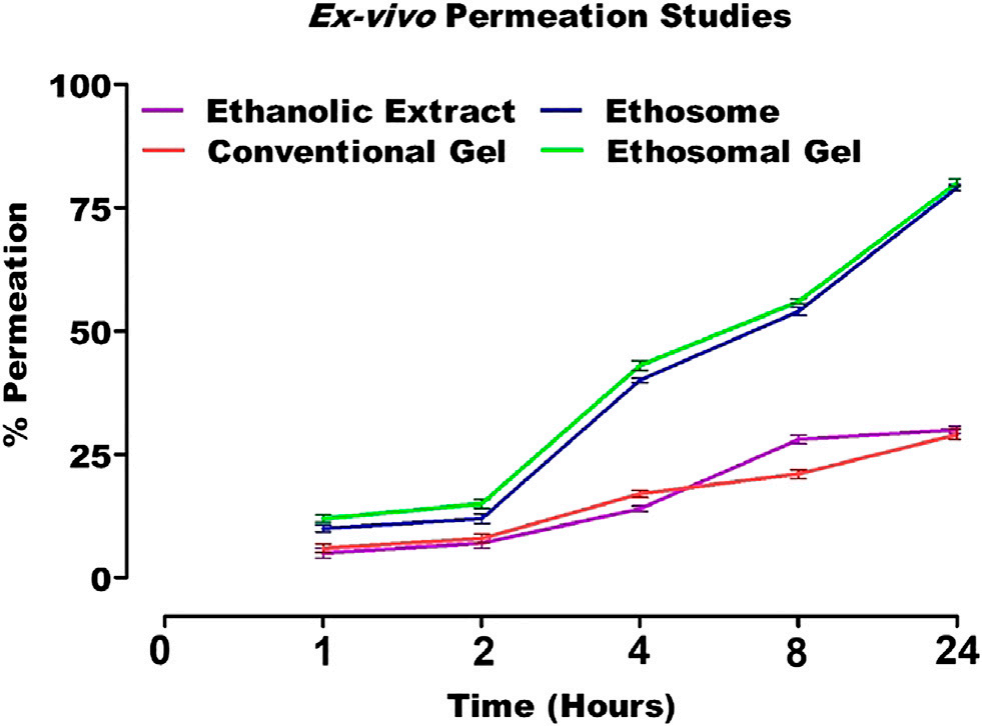
*In vitro* release profile of AM loaded formulations.

Among all formulations, the amount of drug released from ethosomes vesicles and topical gel containing herbal extract were significantly higher as compared to conventional hydro alcoholic extract and conventional gel (Figure 11). This may be due to the presence of ethanol in the formulation as it provides flexibility to the vesicles of dispersion as well as rapid penetration deep into skin layers. The presence of phospholipid may be another factor for efficient penetration as the partition coefficient of lipid is equivalent to the lipid in the stratum corneum. The presence of propylene may also facilitate easy penetration deep into the skin layer (Garg et al., 2016; Chourasia et al., 2011).

**FIGURE 12:**
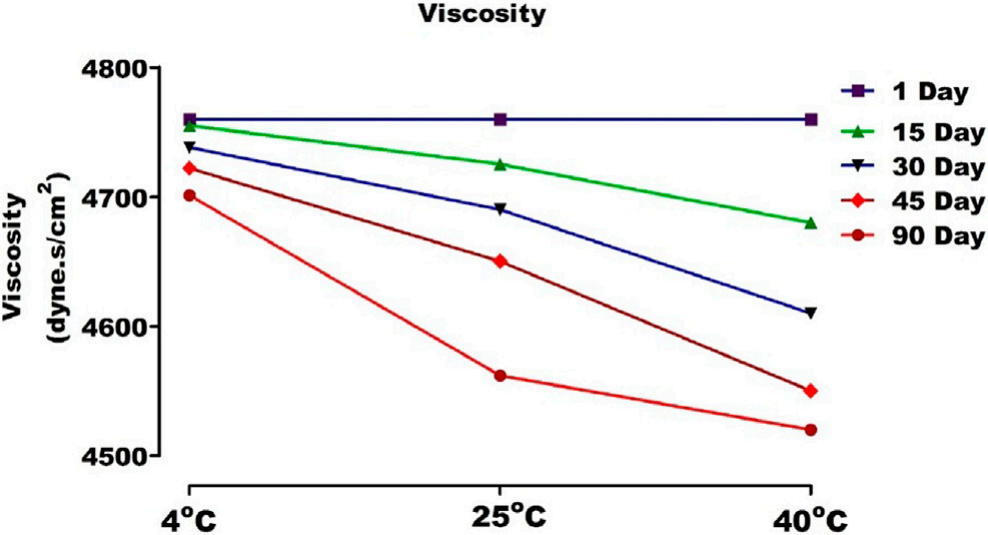
Viscosity of topical gel containing AM extract at different temperature.

With the increase of temperature, the molecular vibrations increased, and ionization became more rapid, resulting in a drop in pH. This may be due to a loss in polymer entanglement at high temperatures. However, this change was not drastic. The viscosities of gel were between 4,760 and 4,520 cP **(** Figure 12 **)**. The results of stability studies indicated that viscosity decreases with the increase in temperature. It can thus be concluded that carbopol based ethosomal gel is thermally stable (Anwar et al., 2017).

## Conclusion

The results of the study concluded that the ethosomal carrier system could be efficient for herbal extracts. A stable topical gel containing nanoethosomes filled with AM extract increased the skin penetration compared to conventional gel and could be a promising product for new researchers.

## Data Availability

The original contributions presented in the study are included in the article/Supplementary Material, further inquiries can be directed to the corresponding author.
