# Supplementary material for: Development, Characterization and Stability Evaluation of Topical Gel Loaded With Ethosomes Containing Achillea millefolium L. Extract
Source: Front Pharmacol. 2021 Apr 12;12:603227. doi: 10.3389/fphar.2021.603227 (PMC8074965; doi:10.3389/fphar.2021.603227)
Supplement: Supplementary file 1 [file datasheet1.pdf]

Centre for Biodiversity & Conservation
Shah Abdul Latif University, Khairpur (Mir's), Sindh-Pakistan

Phone # +92 (243) 9280350

Shah Abdul Latif University, Khairpur
CBC
Centre for Biodiversity & Conservation
مختلف حیاتیاتی تحفظ جو مرکز

CBC/SALU/Khp.482, Dated: Tuesday, 12 March 2019

With reference to information sent by Dr Haji Muhammad Shoaib, Assistant Professor, Department of Pharmacy, Islamia University, Bahawalpur. The nine plant species are verified & identified with the help of different floras and collection available in Herbarium of Centre for Biodiversity and Conservation, SALU. However, the final floral accession number will be allotted after preparation of herbarium specimen.

Plant species

1. Botanical Name: *Achillea millefolium* L.
   Common name: Barijasif
   Family: Asteraceae
2. Botanical Name: Butea superba Roxb.
   Common name Gultaissu
   Family: Fabaceae
3. Botanical Name: Citrullus lanatus (thumb.) Matsum. & Nakai
   Common name: Tarbuz
   Family: Cucurbitaceae
4. Botanical name: Myrciaria dubia (Kunth) Mc vaugh
   Common name: camu camu
   Family: Myrtaceae
5. Botanical name: Berberis vulgaris L.
   Common name: barberry
   Family: Berberidaceae
6. Botanical name: Arctostaphylos uva-ursi (L.) Spreng.
   Common name: kinnikinnick
   Family: Ericaceae
7. Botanical name: Benincasa hispida (Thunb.) Cogn.
   Common name: White Gourd
   Family: Cucurbitaceae
8. Botanical name: Ocimum tenuiflorum L.
   Common name: Tulsi
   Family: Lamiaceae
9. Botanical name: Momordica charantia L.
   Common name: Bitter melon
   Family: Cucurbitaceae

Dr. Mumtaz Ali Saand
Incharge / Director
Centre for Biodiversity & Conservation,
SALU, Khairpur (Mir's)
